# Conceptual competence in psychiatric training: building a culture of conceptual inquiry

**DOI:** 10.1192/bjb.2024.12

**Published:** 2025-04

**Authors:** Awais Aftab, John Z. Sadler, Brent M. Kious, G. Scott Waterman

**Affiliations:** 1 Case Western Reserve University, Cleveland, Ohio, USA; 2 University of Texas Southwestern Medical Center, Dallas, Texas, USA; 3 University of Utah, Salt Lake City, Utah, USA; 4 University of Vermont, Burlington, Vermont, USA

**Keywords:** Philosophy, education and training, bioethics, clinical practice, competence

## Abstract

Building a culture of conceptual inquiry in psychiatric training requires the development of conceptual competence: the ability to identify and examine assumptions that constitute the philosophical foundations of clinical care and scientific investigation in psychiatry. In this article, we argue for the importance of such competence and illustrate approaches to instilling it through examples drawn from our collective experiences as psychiatric educators.

‘The task of philosophy, often a difficult and painful one, is to extricate and to bring to light the hidden categories and models in terms of which human beings think, to reveal what is obscure or contradictory in them, to discern the conflicts between them that prevent the construction of more adequate ways of organising and describing and explaining experience [ … ] and then, at a still ‘higher’ level, to examine the nature of this activity itself … ’ (Isaiah Berlin)^1^

Let's consider a capsulised vignette in the context of clinical supervision in which conceptual issues related to diagnosis and treatment arise as pertinent topics of discussion. Your core trainee, Dr Stevens, comes to present to you a patient, Mr Miller, she has just examined for the first time following an instance of self-harm. After describing the events that preceded Mr Miller's admission, Dr Stevens proceeds to enumerate the diagnostic features by which depressive episodes are defined in the ICD (major depressive disorder in the DSM), indicating for each one whether her examination of Mr Miller demonstrated it to be present or absent. You ask her why she is organising her thinking about Mr Miller in this way, and she replies that it is important to determine whether Mr Miller's behaviour occurred in the context of a ‘real mental disorder’ or whether it was more a reflection of the problems he has been experiencing in his life. You ask her to explain this distinction and she indicates that the importance of aetiology is axiomatic in medicine and that in this case she wishes to know whether Mr Miller's self-harm was ‘caused by a depressive disorder’ or, alternatively, ‘by adverse life events’. Moreover, Dr Stevens continues, treatment choices are guided by such understandings such that a depressive disorder, presumed to reflect ‘abnormal brain biology’, should respond favourably to pharmacotherapy, whereas problems that are ‘psychological’ in nature are better addressed through psychotherapy. You thank her for describing her thought processes as they relate to Mr Miller's condition and begin to examine critically the assumptions that underlie them, along with the questions those assumptions raise and the ways in which those assumptions might run into problems.^2^ You and Dr Stevens are engaging in conceptual inquiry, the outcome of which should augment both her conceptual competence and the quality of Mr Miller's care.

‘Conceptual competence’ (Box 1) involves the transformative awareness of the ways by which background philosophical assumptions influence clinical care and scientific research.^2^ In this article, we will make a case for the importance of conceptual competence by highlighting both the ubiquity of assumptions that guide psychiatric practice and the necessity of examining them critically. We will then describe a number of ‘entry points’ that represent categories of opportunity for weaving conceptual discussions into the various contexts of psychiatric training, from on-the-fly clinical supervision (as above) to didactic seminars. Finally, we will explore the application of the principles of conceptual competence to teaching psychiatric ethics with a case-based approach. Our goal is to encourage and promote a culture of conceptual inquiry in psychiatric training that meaningfully complements other forms of competence – including clinical, cultural, and structural competence – that psychiatric training aims to instil.

Box 1 ‘Conceptual competence’: a question of language

We recognise that the expression ‘conceptual competence’ reflects the increasing use of what an anonymous reviewer described as ‘managerial language’ in medical education. While we share an aversion to empty, formulaic, bureaucratic/managerial language and its consequences, we also must contend with the fact that the current landscape of medical education revolves around the notion of competence. The Accreditation Council for Graduate Medical Education (ACGME) in the USA refers to competencies, while the General Medical Council and Royal College of Psychiatrists in the UK refer to the analogous concept of ‘professional capabilities’. Competencies such as ‘cultural competence' and ‘structural competence' are widely discussed in the medical education literature. By referring to ‘conceptual competence' we are speaking the language of our time and, by doing so, are seeking to ensure that the importance of philosophy in psychiatric education is visible to educators.

## Implicit assumptions in psychiatry

Implicit assumptions play a fundamental role in shaping our understandings of the world,^3^ including psychiatric conditions and psychological suffering. These assumptions are part of the philosophical underpinnings of our scientific and clinical practices.^2,4^ They relate to matters such as what we find desirable and preferable (values), our beliefs about the fundamental natures of psychiatric conditions and psychological suffering (ontology), and about the sorts of knowledge we can obtain about psychiatric phenomena and how it can be obtained (epistemology). These philosophical assumptions are so deeply ingrained that we rely on them implicitly to guide and justify our scientific and clinical work. Although it is not possible or even desirable to eliminate these assumptions completely – all empirical observations are theory-laden, as philosophers of science often point out^5^ – we can make conscious efforts to bring them to light and make them explicit.

By making implicit assumptions explicit, we open the door to scrutiny, allowing us to challenge and, if necessary, replace our assumptions.^6,7^ Philosophical assumptions and biases are acquired from a variety of sources, including folk intuitions, education, professional training and practice, and disciplinary traditions. All of these factors contribute to the establishment of scientific paradigms that influence and justify our scientific and clinical practices.

A prominent example comes from the history of psychiatric descriptive classifications, where even purportedly ‘atheoretical’ frameworks such as DSM-III were deeply embedded in neo-Kraepelinian assumptions.^8,9^ Those assumptions included the existence of discrete disease entities and the expectation that external validators identified through research would ‘converge’ on the characterisation of those entities.^10^ Such beliefs shaped the trajectory of psychiatric research over the course of subsequent decades, even if the DSM project later disavowed such inferences.

To address the potential confusion arising from these implicit assumptions – an example of which is cited above, but which are likely to differ across individuals and across time – it is essential to encourage critical reflection within our scientific and clinical communities. By fostering open dialogue and acknowledging the influences of these philosophical assumptions, we can strive for more comprehensive and accurate ways of understanding and addressing psychiatric conditions. This self-awareness and willingness to reevaluate our assumptions will, one hopes, lead to advances in the field and ultimately to improved patient care and outcomes.

We contend that a neglect of conceptual competence has led to a state of conceptual impoverishment of mainstream psychiatric practice. We do not mean this to imply that psychiatry as a subject lacks conceptual richness but rather it is our view that many practitioners have lost sight of that. Such a view has been echoed by many prominent psychiatric leaders, including Nancy Andreasen, who has lamented the ‘death of phenomenology’ in America.^11^ The reification of DSM and ICD constructs, including the misconception that diagnostic criteria constitute rather than index psychiatric conditions,^12^ has limited the potential richness and flexibility of psychiatric understanding that is offered to us by approaches that take neuroscientific, phenomenological and narrative perspectives seriously.^13^ Additionally, a tendency towards biological reductionism has narrowed the scope of psychiatric research, often overlooking the multifaceted nature of psychiatric phenomena. The biopsychosocial model, although well-intentioned and clinically useful, has been said to lack a philosophical account of causal interactions^14^ and to have generated confusion about the meaning and goals of holism in medicine.^15–18^ Diagnostic inflation has resulted in a steady expansion of the domain of mental illness, raising questions about when difference and distress become disorders,^19^ and has led to a common grievance that aspects of ordinary life have been unnecessarily medicalised or pathologised.^20^ Equally concerning is the lack of sufficient representation of patient and ex-patient perspectives in psychiatric practice and science, resulting in inadequate understandings of their complex needs and preferences.^21^ This has been driven in part by a neglect of philosophical frameworks, such as standpoint epistemology and social objectivity, that justify the inclusion of lived experience in science.^22,23^

The conceptual impoverishment of psychiatric practice and training has taken place despite the existence of a rich and growing body of conceptual and philosophical work. The key to rebuilding a culture of inquiry lies in recognising and cultivating conceptual competence within healthcare.^2^ As noted above, conceptual competence involves the transformative awareness of how background philosophical assumptions – held by clinicians, patients and members of society at large – shape various aspects of clinical practice, research and education.

Development of conceptual competence entails four essential elements. First is identification and articulation of otherwise-implicit conceptual assumptions and corresponding questions that underpin clinical interactions and diagnostic practices. Second is acquisition of a philosophical vocabulary and familiarity with relevant arguments and frameworks by which to examine, rigorously and systematically, those conceptual assumptions. Third is engagement in organised philosophical discourse to evaluate the merits of different responses to conceptual questions relevant to psychiatric practices. And finally comes cultivation of conceptual humility, recognition of the tentative nature of scientific and philosophical formulations, and appreciation of the value of pluralism in our explanatory thinking.

Teaching conceptual competence requires active, dynamic exchanges of ideas. Conceptual discourse is not a matter of passive acquisition of knowledge. Rather, it demands critical engagement and practice. Trainees must be equipped to apply conceptual tools and examine responses to conceptual questions in their training environments. Conversations and debates found in the academic literature can serve as valuable aids in teaching philosophical discourse.

It is essential to acknowledge that conceptual problems are seldom definitively solved or settled. Developing conceptual humility involves recognition of the complexity of the topics involved and acknowledgement that even with our best efforts, some questions are likely to remain unresolved. Understanding that all philosophical frameworks have inherent limitations is integral to cultivating this virtue.

Several elements of the 2022 core psychiatry training curriculum by the Royal College of Psychiatrists (accessible at www.rcpsych.ac.uk/training/curricula-and-guidance/curricula-implementation/curricula-documents-and-resources) are relevant to conceptual competence. Understanding of the development of diagnostic concepts, the development of the profession, historical relationships between psychiatry and society, and a person-centred holistic approach to mental disorders are noted in the curriculum as key capabilities. These are all domains in which understanding requires grappling with implicit as well as explicit conceptual assumptions. The core curriculum also expects trainees to demonstrate an understanding of factors that contribute to complexity and uncertainty in psychiatric practice. This is again relevant to conceptual competence, in particular to the element of ‘conceptual humility’. The framework of conceptual competence can therefore be of value to educators in developing these key capabilities.

In the UK and global contexts, values-based practice (VBP)^24^ is an admirable example where we can see elements of conceptual competence in action. VBP is a clinical skills-based approach to working with complex and conflicting values in healthcare. VBP encourages the development of skills that identify implicit values at play in any clinical interaction. It offers a rigorous and scholarly framework by which to consider these values in clinical and academic contexts. VBP emphasises the necessity of interdisciplinary dialogue and recognises the humility needed to navigate complex and conflicting values. (The Collaborating Centre for Values-based Practice in Health and Social Care at St Catherine's College, Oxford (valuesbasedpractice.org), is an exceptional resource for learning more about this approach.)

## Entry points in teaching conceptual competence

This section considers some examples of teaching conceptual competence in common psychiatric training settings. As introduced above, the first element of teaching conceptual competence (TCC) is developing trainees’ awareness of philosophical assumptions, which frequently entail unrecognised questions, ambiguities and conflicts.

TCC requires a shift in the educator's awareness. One element of that involves movement away from the diagnosis–treatment mode of clinical reasoning and towards use of clinical problems as basic units of clinical reasoning.^25^ Educators must also recognise the core concept of ‘reduction’ in clinical science and practice. Reduction is the notion that every framework we use requires us to pay attention to some aspects of phenomena and to neglect other aspects. This means that as we shift our frameworks (e.g. aetiopathogenesis framed in neurobiological terms, psychotherapeutic theories, cultural contexts of human distress, etc.), the observational data themselves change.^26^ Viewing cases through different ‘lenses’ entails conceptual humility, as introduced above. Educators must also recognise that conceptual matters are often tacit, or taken for granted, and that attentional shifts towards making them explicit contribute to improved practice through making such issues teachable.

We identify and outline briefly below four broad categories of opportunity for TCC suitable to different training and clinical settings: (a) ambiguities in meaning; (b) conflicts between imperatives; (c) inexplicit intuitions; and (d) faulty clinical reasoning.

### Ambiguities in meaning

A central technique in philosophical practice, and TCC, is distinguishing *word* from *concept*. Meanings in language are ambiguous because one word may correspond to multiple concepts. The converse – multiple words pointing to single concepts – also occurs. Recognising word/concept ambiguities is a fundamental TCC skill. For example, a patient states ‘I'm paranoid’. One word (‘paranoid’), many concepts: the patient could be referring to delusions of persecution, hypervigilance resulting from trauma, anxiety, intoxication from cannabis, etc.

### Conflicts between imperatives

Conflicts between imperatives (important things to consider or do) are familiar in clinical ethics (see below), where we often encounter conflicts between values (preserving autonomy by not admitting a refusing patient to hospital) and beneficence (assuring safety by arranging involuntary admission). Conflicts between imperatives can also be practical in nature, as in prioritisation of services (housing-first for an undomiciled alcohol-dependent individual, versus sobriety-first). A third category of conflict between imperatives is epistemic: adhering to, versus deviating from, professional treatment guidelines in a difficult case.

### Inexplicit intuitions

We know that only a small portion of our clinical judgements are based on the ‘evidence base’. The rest of clinical judgement is driven by patient values, practical and ethical constraints, and various intuitions that we often take for granted. To ‘unmask’ these intuitions is to make them explicit and then subject them to critical reflection. For example, a man with a history of depression, now remitted, complains of ‘anxiety’ related to an upcoming evaluation for an elevated prostate-specific antigen concentration. How do we determine what is normative versus pathological anxiety, potentially warranting a change of treatment, in such a context?

### Faulty clinical reasoning

Although the conceptual issues in clinical reasoning are complex, we can mention a few common examples here. ‘Premature closure’ is perhaps the most common among trainees (residents). For example, a trainee assumes that a patient with prominent trauma-related symptoms is unlikely to respond to medication treatment without psychotherapy, which the patient does not wish to pursue. The resident has generated a patient standoff though this premature closure. A second example is ‘diagnosis by treatment response’. A trainee, for example, has concluded that a patient with prominent mood dysregulation is ‘bipolar’ because she improved on aripiprazole. Diagnosis by treatment response is flawed conceptually because, among other reasons, the correspondence between diagnostic categories and treatment responses is far from one-to-one. Our third example of faulty reasoning is the classic fallacy, *post hoc ergo propter hoc* (after this, therefore because of this). For example, a trainee accepts without further investigation a patient's complaint of worsening anxiety shortly after starting an initial dose of lamotrigine, noting this as a ‘failed’ medication trial due to an adverse response.

## Teaching conceptual competence in addressing ethical dilemmas

In psychiatric ethics, conceptual competence involves the ability to apply complex ethical principles in the approach to clinical cases that represent ethical dilemmas. The US Accreditation Council for Graduate Medical Education's ‘Professionalism’ Milestones for psychiatry recognise five levels of competence in clinical ethics: (1) knowledge of core ethical principles; (2) analysing straightforward situations using ethical principles; (3) analysing complex situations using ethical principles and knowing when help is needed; (4) using appropriate resources for managing and resolving ethical dilemmas as needed, such as ethics and risk management consultations; and (5) identifying and addressing systemic factors that contribute to ethical problems.^27^ Although these competencies are clearly important, the complexity of ethical dilemmas faced by psychiatrists in training and practice, and the unique problems faced in psychiatry,^28^ demand even greater skills. Psychiatrists and psychiatry residents should be able to recognise that many ethical dilemmas are fundamentally complex, in the sense that they involve competing ethical considerations and perspectives that may be irreconcilable. Approaching such ethical dilemmas involves accepting this complexity while working towards practical responses.^29^

In the programme run by one of us (B.M.K.), a case-based approach to ethics education is employed that aims to help fourth-year residents embrace ethical complexity. Through its encouragement of virtuous self-awareness and humility, this method combines recommendations that ethics education should focus on development of virtuous clinicians^30^ with claims that it should focus on knowledge and application of ethical principles.^31^

For each teaching session, residents provide descriptions of difficult cases in advance, identifying some salient ethical difficulties they involve. Faculty members review the cases, develop learning objectives and provide accessible philosophical readings responsive to those objectives. Within each session, faculty members explain some of the philosophical ideas represented in the readings and then lead case discussions that identify gaps in the residents’ ethical assessments. This promotes recognition of complexity and fosters more sophisticated, nuanced analyses.

This process emphasises that making an ethically good decision might not mean reaching the *right* conclusion but, rather, following a process that is thoughtful and inclusive, admits multiple competing individual and cultural perspectives, and acknowledges uncertainties at many levels.^32^ Residents are encouraged to observe how ethical analysis is sensitive to facts that might be unknown or poorly defined. They are asked to consider how competing moral principles (such as respect for autonomy and beneficence^33^) might simultaneously bear on a case and that there might be no guide to balancing them.^34^ They see how even when a principle is relevant, its correct application is subject to debate, as it depends on concepts that are themselves ambiguous.^35^ The intended result of this process is that the residents discussing a case identify a reasonable plan of action, while acknowledging its limitations.

For illustration, consider a composite case. The psychiatry consultation team is called by the surgery service to evaluate a man who attempted to castrate himself in prison. He did not succeed but his injuries threaten the viability of the affected tissues. The man was incarcerated for sexual offences and hoped that the castration would demonstrate remorse, make him less likely to reoffend, and promote the possibility of clemency. He wants the surgical team to complete an orchiectomy, but they refuse. The consultation team interviews him and concludes that he has no diagnosable mental illness and has full decision-making capacity.

To help guide the residents’ discussion of the case, philosophical readings are provided that problematise the concept of autonomy.^36–38^ The residents are invited to consider how structural factors like the individual's incarceration can affect autonomy. They are urged to monitor their own responses to him, as the case is likely to produce strong emotional reactions, such as shock at the man's self-injury or disgust at his crime, that could colour ethical judgements.^39^ The residents decide that there is no clear conclusion. Still, they identify positives and negatives for each potential course of action. Following the individual's wishes would respect his autonomy, probably reduce short-term medical risks and conceivably reduce the risk of reoffending. On the other hand, it might make him worse off in the long run, and would involve complicity in an act that seemed coerced and unreasonable.

## Conclusion

Psychiatry is complex in a number of ways and thus the development of sufficient competence to practise it well is arduous. One category of competence to which we wish to call greater attention – owing to the signs of its relative neglect in psychiatric training, clinical care and science – is conceptual competence. We believe that psychiatrists should recognise (and question) the myriad philosophical assumptions that implicitly underpin their everyday work. There are several ‘entry points’ for teaching this awareness of the influence of background philosophical assumptions on clinical care across training contexts, and such conceptual competence in addressing ethical dilemmas can be instilled during residency. We hope that clinical educators take up our appeal to establish conceptual competence as a consensus desideratum of psychiatric training. The credibility of the profession and the well-being of the people it seeks to understand and help depend on it.

## About the authors

**Awais Aftab**, MD, is Clinical Assistant Professor of Psychiatry in the School of Medicine at Case Western Reserve University in Cleveland, Ohio, USA. **John Z. Sadler**, MD, is Professor of Psychiatry and the Daniel W. Foster, MD, Professor of Medical Ethics at the University of Texas Southwestern Medical Center in Dallas, Texas, USA. **Brent M. Kious**, MD, PhD, is Assistant Professor of Psychiatry, Adjunct Professor in the Center for Bioethics and Health Humanities, and Adjunct Professor in the Department of Philosophy at the University of Utah in Salt Lake City, Utah, USA. **G. Scott Waterman**, MD, MA, is Professor of Psychiatry Emeritus in the Larner College of Medicine at the University of Vermont in Burlington, Vermont, USA.

## Data Availability

Data availability is not applicable to this article as no new data were created or analysed in this study.
